# Continuous leaky-wave scanning using periodically modulated spoof plasmonic waveguide

**DOI:** 10.1038/srep29600

**Published:** 2016-07-12

**Authors:** Gu Sheng Kong, Hui Feng Ma, Ben Geng Cai, Tie Jun Cui

**Affiliations:** 1 State Key Laboratory of Millimetre Waves, School of Information Science and Engineering, Southeast University, Nanjing 210096, China; 2 Synergetic Innovation Center of Wireless Communication Technology, Southeast University, Nanjing, 210096, China

## Abstract

The plasmonic waveguide made of uniform corrugated metallic strip can support and guide spoof surface plasmon polaritons (SSPPs) with high confinements. Here, we propose periodically-modulated plasmonic waveguide composed of non-uniform corrugated metallic strip to convert SSPPs to radiating waves, in which the main beam of radiations can steer continuously as the frequency changes. To increase the radiation efficiency of the periodically-modulated plasmonic waveguide at the broadside, an asymmetrical plasmonic waveguide is further presented to reduce the reflections and realize continuous leaky-wave scanning. Both numerical simulations and experimental results show that the radiation efficiency can be improved greatly and the main beam of leaky-wave radiations can steer from the backward quadrant to the forward quadrant, passing through the broadside direction, which generally is difficult to be realized by the common leaky-wave antennas.

Spoof surface plasmon polaritons (SSPPs) can be supported and propagated on the metal surfaces decorated by slits, holes, or blocks in the microwave, terahertz, and far-infrared frequencies^1^,^2^,^3^,^4^,^5^,^6^,^7^,^8^,^9^, whose characteristics are similar to surface plasmon polaritons (SPPs) in the optical region. The SSPPs are usually bounded on the decorated metal surfaces tightly, propagating parallel to the surfaces and decaying exponentially vertical to the surfaces. More recently, a kind of ultrathin corrugated plasmonic waveguides has been proposed to support and propagate SSPPs with high efficiency^10^. Compared to the conventional two-conductor transmission lines, this plasmonic waveguide can transmit the signals by only using single conductor, which has advantages in designing integrated circuits. In order to integrate such plasmonic waveguides with traditional microwave circuits, the matching transitions between the plasmonic waveguides and traditional microstrip or co-planar waveguide structures have also been proposed to make smooth conversions between SSPPs and spatial modes^11^,^12^,^13^, and the high-order modes of such plasmonic waveguides also haves been studied^14^. Using such plasmonic waveguides, a number of passive and active functional devices have been proposed, such as SPP filter^15^, SPP amplifier^16^, SPP coupler^17^, and others^18^,^19^,^20^. However, all above mentioned devices are based on the characteristics of strong confinement and high-efficiency transmission of SPPs on the plasmonic waveguide. This is because of the slow-wave feature of SSPPs, whose *k* vectors along the propagating direction are larger than *k*_0_ in free space. In ref. 21, a plasmonic waveguide was proposed to combine with metasurface to make radiations, in which plasmonic waveguide is only used as feeding line and radiation part is metasurface. Hence, if the radiating devices based on plasmonic waveguide are required in applications, such as antennas, the surface waves of the plasmonic waveguide must be converted to the fast wave with *k* < *k*_0_.

Leaky-wave radiations are usually generated by leaking the electromagnetic energy gradually over a structure, which can be called as leaky-wave antennas^22^. The radiated beams can scan in the space as frequency changes in operation. Generally, there two types of leaky-wave antennas: uniform and periodic^23^. The uniform leaky-wave antennas such as slot waveguides can only scan in forward quadrant, which cannot approach broadside too closely. The periodic leaky-wave antennas such as periodically-loaded dielectric waveguides can scan in both forward quadrant and backward quadrant, but an “open stop band” occurs around broadside due to mode coupling with low radiation efficiency^23^,^24^,^25^. Hence, it is always a challenge for leaky-wave antenna to obtain high efficient radiation around broadside, and only a few works have been proposed to improve the radiation of leaky-wave antennas at broadside^26^,^27^,^28^. In early time, T. Itoh has proposed that the composite right/left-handed (CRLH) transmission line (TL) can achieve high efficient radiation at broadside under the balanced condition, which requires complicated sub-wavelength unit cells to construct artificial series capacitance and shunt inductance for balancing series and shunt TL impedances^26^,^27^. More recently, M. Memarian and G. V. Eleftheriades proposed a kind of Dirac Leaky-wave antenna (DLWA) made up of a photonic crystal to make high efficient radiation at broadside due to the closed Γ-point bandgap of the Dirac PC^28^, which is designed based on a dielectric-loaded rectangular waveguide. More recently, a one-dimensional (1D) planar corrugated surface with infinite thickness and a cylindrical corrugated surface have been proposed to radiate surface waves^29^, but these bulky structures are not only difficult to be integrated with conventional circuits but also helpless to improve the radiation efficiency at broadside.

In this paper, we present periodically-modulated plasmonic waveguides composed of two-side corrugated metallic grooves to convert SSPPs to radiating waves. The periodic modulation introduces infinite space harmonics *k*_*N*_ (*N* = 0, ±1, ±2, …), in which the space harmonics with negative *N* can be fast with *k*_*N*_ < *k*_0_ under suitable conditions. In practice, the *N* = −1 space harmonic is usually chosen to obtain single radiation beam, which can be achieved by modulating the groove depths of the plasmonic waveguide periodically. The simulation results show that the periodically-modulated plasmonic waveguide can convert SSPPs to radiating waves, in which the radiation pattern along azimuth is omnidirectional, while the radiation pattern along elevation can be steered as the frequency changes. However, we find that the radiation efficiency is reduced dramatically near the broadside radiation, which is due to large reflections of energy back to the feeding line^23^. To improve the radiation efficiency at the broadside direction, we simply stagger the periodically modulated grooves on both sides of the plasmonic waveguide with a phase displacement of π/2 along the propagating direction, so that the reflections generated by each pair unit cells on both sides are cancelled to each other. Different from the symmetrical case, the radiation pattern of the asymmetrical plasmonic waveguide is not omnidirectional along the azimuth, which is due to the enhancement and cancellation of radiating energies in forward and backward radiations, respectively. As frequency changes, the leaky-wave radiation can steer in continuous way, from the backward quadrant to broadside, then through the broadside to the forward quadrant. The measurement results have good agreements with numerical simulations, which have potential applications in integrated plasmonic circuits and antennas.

## Results

### Theoretical analysis

**Figure 1:**
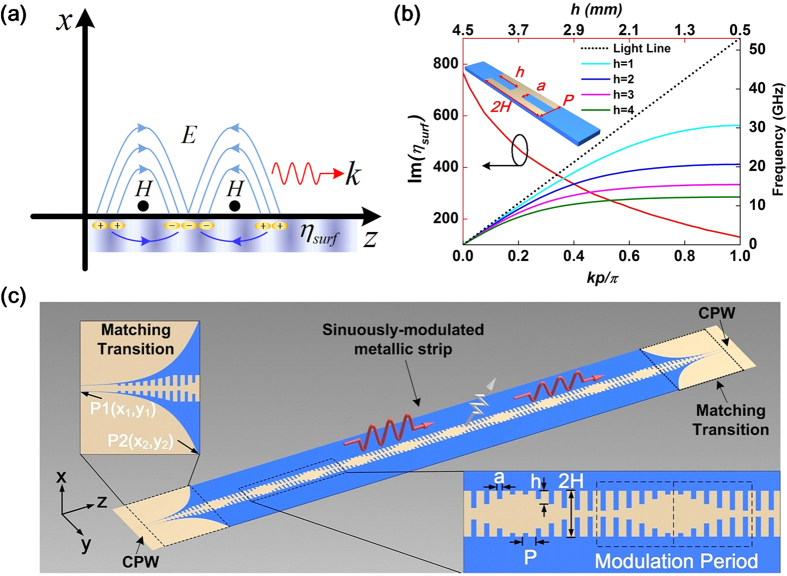
The demonstrations of SPPs and periodic-modulated SSPPs. (**a**) The schematic of electromagnetic-field distribution of SPPs. (**b**) The dispersion curves and surface impedance of symmetrical two-side metallic grooves with different groove depths *h*. (**c**) The schematic of sinusoidally-modulated plasmonic waveguide using the corrugated metallic strip.

The SPPs are bounded on the boundary of two different dielectrics, which propagate parallel along the interface and decay exponentially in the direction vertical to the interface, as demonstrated in Fig. 1(a). According to the Maxwell Equations, the electromagnetic fields in the upper space should satisfy the following equations


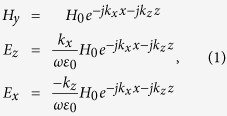


For lossless media, *k*_*z*_ = *β*_*z*_ is the propagating constant along the +*z* direction (*β*_*z*_ is a positive real number), and *k*_*x*_ = −*jα*_*x*_ is the propagating constant along the +*x* direction (*α*_*x*_ is also a positive real number). The relationship between the *k*_*z*_ and *k*_*x*_ can be written as 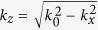, in which *k*_0_ is the wavenumber in free space. According to Eq. (1), the surface impedance along the +*z* direction is calculated as


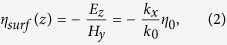


where *η*_0_ is the wave impedance in free space. Hence the relationship between the propagating constant along the surface (*k*_*z*_) and surface impedance (*η*_*surf*_) along the +*z* direction is obtained


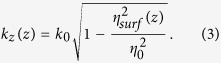


SSPPs at lower frequencies have similar characteristics to SPPs in the optical frequency. The corrugated metallic strip is a typical plasmonic waveguide, which can support and propagate SSPPs in the microwave and terahertz frequencies. Figure 1(b) shows the dispersion curves of one unit cell of the corrugated metallic strip by varying groove depth, illustrating that the wavenumbers *k*_*z*_ along the propagating direction become larger as the groove depth (*h*) increases with *k*_*z*_ > *k*_0_. The plasmonic waveguide is designed by using printed circuit board (PCB) of F4B with relative permittivity of 2.65 and loss tangent of 0.001, and the thickness of the substrate is chosen as *t* = 0.5 mm. The dimensions of unit cell shown in Fig. 1(b) are *H* = 5 mm, *a* = 1.13 mm, *p* = 2.825 mm, and varied *h*. Generally, SSPPs are slow waves (*k*_*z*_ > *k*_0_) bounded on the surface of the plasmonic waveguide tightly, which cannot be radiated into the free space. In order to convert SSPPs to radiating waves, we modulated the surface impedance of the plasmonic waveguide sinusoidally by changing the depths of metal grooves obeying the following equation


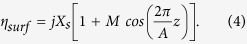


in which *X*_*s*_ is average surface reactance, *M* is degree of modulation, and *A* is modulation period. The model of the sinusoidally-modulated plasmonic waveguide is demonstrated in Fig. 1(c).

The modulated plasmonic waveguide can introduce infinite space harmonics^18^, whose phase constant *k*_*N*_ can be calculated as


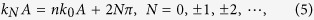


where *k*_0_ is the phase constant in free space, and *n* is an effective surface refractive index (*n* ≥ 1). Equation (5) shows that the space harmonics are all slow waves for *N* ≥ 0 with *k*_*N*_ > *k*_0_. But once *N* ≤ −1, the space harmonics can be fast with |*k*_*N*_| < *k*_0_ under suitable conditions. According to Eq. 5, if *N* ≤ −2 space harmonics are designed to be fast and radiated, then *N* = −1 space harmonic usually is avoidable to be fast and radiated. Hence, in order to obtain a single radiated beam, *N* = −1 space harmonic is usually chosen in practice. We assume that the angle between the radiation beam and the +*z* direction is *θ*, as shown in Fig. 1(c). Hence the relationship between *k*_−1_ and *θ* can be written as


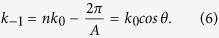


To make *N* = −1 space harmonic be fast wave, the condition of |*k*_−1_| < *k*_0_ must be guaranteed, and hence the range of effective surface refractive index *n* as determined as


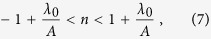


where λ_0_ is the wavelength at the working frequency *f*_0_. According to Eqs (3) and (4), the relationship between *n* and *X*_*s*_ is set up as


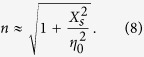


Hence the range of *X*_*s*_ is also fixed according to Eq. (7). Once the parameters *X*_*s*_ and *A* are determined, the modulation parameter *M* should be chosen to satisfy the range of *η*_*surf*_ based on Eq. (4). From Eq. (3), the surface impedance of each unit cell can be calculated as


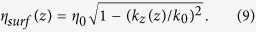


The range of *k*_*z*_ can be determined by changing the groove depth (*h*) of unit cell from the minimum value to the maximum value at the working frequency. Then the range of *η*_*surf*_ can be determined from Eq. (9). According to the unit cell shown in Fig. 1(b), the surface impedance *η*_*surf*_ of the proposed plasmonic waveguide can cover the range from 766 to 130 when *h* varies from 4.5 mm to 0.5 mm at 9.3 GHz.

### Design, simulation and experiment

Figure 1(c) shows a schematic of the designed sinusoidally-modulated plasmonic waveguide, which is composed of two coplanar waveguides (CPWs), two matching transitions, and a sinusoidally-modulated metallic strip. CPWs in two ports are designed with 50 Ω impedance, and two matching transitions between CPWs and the sinusoidally-modulated metallic strip are designed to make a smooth conversion between the spatial waves (supported by CPWs) and SSPPs (supported by the sinusoidally-modulated metallic strip)^11^. PCB used in our design is chosen as F4B with the thickness of 0.5 mm, and the thickness of the printed copper film is 0.018 mm.

According to Eq. (6), the direction of the radiation beam is designable by choosing different *A* and *n*. Here, we design a sinusoidally-modulated plasmonic waveguide arbitrarily to radiate the spatial waves to the direction of *θ* = 55° from the +*z* axis at 9.3 GHz. In our design, the modulation period *A* is chosen as 33.9 mm with 10 periods, in which each period (*p* = 2.825 mm) contains 12 symmetrical metallic grooves with different groove depths *h*, as shown in Fig. 1(b). Once the period *A* and radiation angle *θ* are fixed, then the effective surface refractive index can be calculated as *n* = 1.52 according to Eq. (6). Hence we choose *X*_*s*_ = 1.1*η*_0_ according to Eq. (8). Furthermore, the surface impedance of plasmonic waveguide can be modulated from min(*η*_*surf*_) = 130 to max(*η*_*surf*_) = 766 at 9.3 GHz, when the groove depth *h* is increased from 0.5 mm to 4.5 mm, as the red solid line demonstrated in Fig. 1(b). As a result, from Eq. (4), we choose M = 0.68 so that the surface impedance of the modulated plasmonic waveguide can cover the range of the minimum and maximum *η*_*surf*_ as much as possible to achieve the high-efficiency radiation. Hence the final groove depths (*h*) of the sinusoidally-modulated plasmonic waveguide are determined according to the relationship between the surface impedance *η*_*surf*_ and the groove depth *h*, as the red solid line shown in Fig. 1(b).

**Figure 2:**
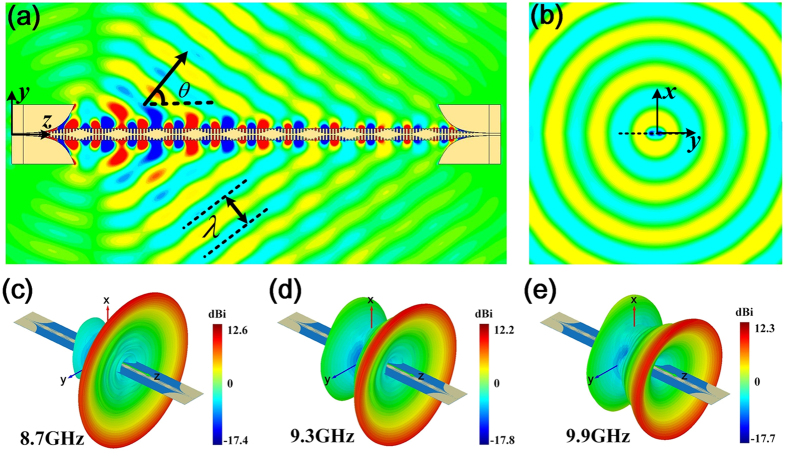
The simulation results of the sinusoidally-modulated plasmonic waveguide. (**a**) The near electric-field distribution in the plane of *x* = 0, which clearly shows that SSPPs are converted to radiating waves (leaky waves) with radiation angle *θ*. (**b**) The near electric-field distribution in the *xoy* plane, showing that the waves are radiated omnidirectionally in the azimuth direction. (**c**–**e**) The simulated 3D far-field radiation patterns at 8.7 GHz, 9.3 GH and 9.9 GHz with radiation angles of 63.9°, 54.9° and 43.7°, respectively.

Figure 2(a) presents the near-field distribution of *E*_*z*_ by using the commercial software, CST Microwave Studio, in the *yoz* plane with *x* = 0, which clearly shows that the energy of surface waves is decreased gradually and radiated to the free space with an angle of *θ* = 55° when SSPPs propagate along the +*z* direction. Figure 2(b) shows the near-field distribution of *E*_*z*_ in the *xoy* plane with *z* = 205 mm, illustrating that the radiation beam along azimuth is nearly omnidirectional around the plasmonic waveguide. The simulated far-field radiation patterns are given in Fig. 2(c–e) at 8.7 GHz, 9.3 GHz, and 9.9 GHz with radiation angles of 63.9°, 55.9° and 43.7°, respectively.

**Figure 3:**
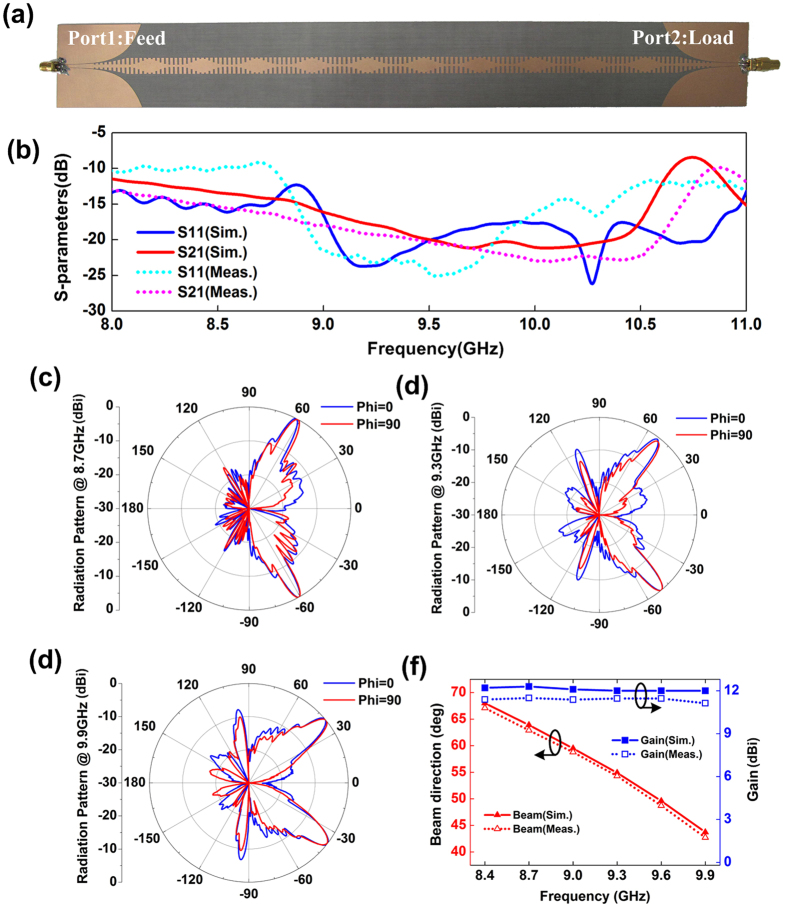
The fabricated sample of the sinusoidally-modulated plasmonic waveguide and the measured results. (**a**) Photograph of the fabricated sample. (**b**) The measured S parameters. (**c**–**e**) The measured far-field radiation patterns of E plane in both *φ* = 0 and *φ* = 90° planes at 8.7 GHz, 9.3 GH and 9.9 GHz with radiation angles of 63.9°, 54.9° and 43.7°, respectively. (**f**) The measured gains and radiation angles at different frequencies.

The final fabricated sinusoidally-modulated plasmonic waveguide is demonstrated in Fig. 3(a), whose measurement S parameters are presented in Fig. 3(b). Both the transmission coefficient *S*_21_ and reflection coefficient *S*_11_ are lower than −10 dB from 8.4 GHz to 10 GHz, which means that the power is radiated to the free space efficiently. We can evaluate the radiation efficiency from the S parameters by 1 − |*S*_11_|^2^ − |*S*_21_|^2^ due to the low loss of the plasmonic waveguide at microwave frequencies. According to Fig. 3(b), the simulated radiation efficiency can be calculated with a minimal of 90% from 8.4 GHz to 10 GHz and maximum of 95% from 9 GHz to 10 GHz. The measured radiation efficiency also can be calculated with 95% from 8.8 GHz to 10 GHz, but which becomes worse with 86% from 8.4 GHz to 8.8 GHz. The worse radiation efficiency in measurement composed to the simulation is caused directly by the large measured *S*_11_, which may be due to the mismatch between the 50 Ω coaxial SMA connectors and CPWs on the two terminals of the plasmonic waveguide in measurement, because the full-wave simulations are carried out by using wave-port setup directly instead of using SMA connectors. Figures 3(c–e) show the measured far-field patterns of E plane in both *φ* = 0 (*xoz*) and *φ* = 90° (*yoz*) planes, and the patterns in both planes have good agreements to each other with main beams directing to 62.9°, 54.3° and 42.7° at 8.7 GHz, 9.3 GHz and 9.9 GHz, respectively. The measured gains and radiation angles at different frequencies are demonstrated in Fig. 3(f), which show that the measured gain is about 11.4 dBi and the radiation angle can be steered from 67.1° to 42.7° as the frequency changes from 8.4 GHz to 9.9 GHz.

**Figure 4:**
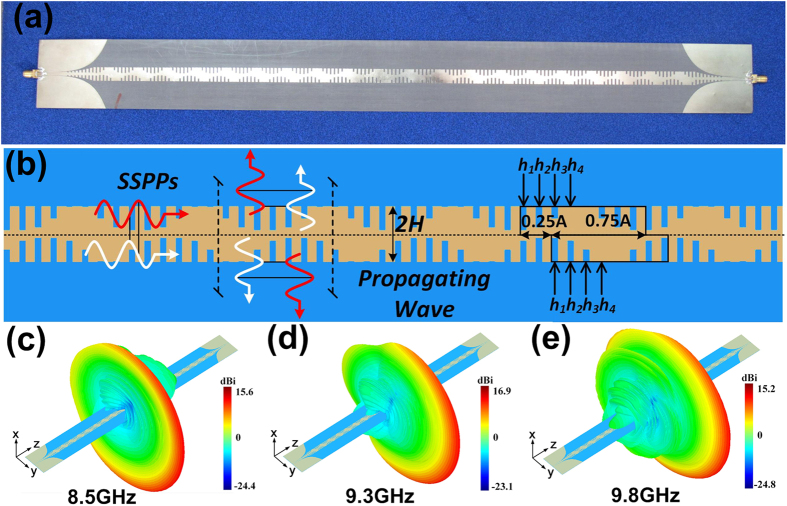
The fabricated sample of asymmetrically sinusoidally-modulated plasmonic waveguide and 3D far-field radiation patterns. (**a**) The photograph of the fabricated sample. (**b**) The detailed structure with different groove depths of *h*_1_, *h*_2_, *h*_3_ and *h*_4_. (**c**–**e**) The simulated 3D far-field radiation patterns at 8.5 GHz, 9.3 GH and 9.8 GHz with radiation angles of 100°, 90° and 82°, respectively.

However, the radiation efficiency near the broadside direction (*θ* = 90°) is decreased dramatically for the periodic structures with large voltage standing wave ratio (VSWR), and the energy in this region is mostly reflected back to the feeding source rather than being radiated, which is called as “open stop band” region^23^. In order to improve the radiation efficiency at broadside, we propose an asymmetrical plasmonic waveguide to cancel the reflections, as shown in Fig. 4(a), in which the periodically modulated grooves on both sides of the corrugated strip have a *λ*/4 offset along the propagation direction. In this way, the reflections generated by upper and lower grooves can be cancelled with each other to obtain highly-efficiency radiation at the broadside direction.

The dimensions of the asymmetrical plasmonic waveguide are *A* = 22.6 mm, *a* = 1.13 mm, 2*H* = 10 mm, *P* = 2.825 mm, *X*_*s*_ = *η*_0_ and M = 0.88. The upper and lower modulated grooves have *λ*/4 offset along the propagation direction with different groove depths in one period of *h*_1_ = 4.3 mm, *h*_2_ = 3.8 mm, *h*_3_ = 2.2 and *h*_4_ = 0.1 mm, as illustrated in Fig. 4(b). The full-wave simulation results of three-dimensional (3D) radiation patterns at 8.5 GHz, 9.3 GHz and 9.8 GHz are demonstrated in Fig. 4(c–e), respectively, which show that the main lobe can steer from backward quadrant (from 8.5 GHz to 9.3 GHz) to forward quadrant (from 9.3 GHz to 9.8 GHz), through the broadside (at 9.3 GHz) exactly. However, we notice that the radiation patterns are not omnidirectional along azimuth any more, in which the power is mostly radiated to the +*y* direction but reduced in the −*y* direction. The reason is that the periodically modulated grooves have *λ*/4 offset along the propagating direction, leading to π/2 phase difference on the same cross section of plasmonic waveguide. Hence the phase difference on any interface of corrugated metallic strip in +*y* and −*y* sides will be 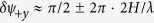 and 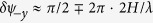. As a result, if the δψ + y → 0, then δψ − y → π, and the power will be superposed in the +*y* direction and cancelled in the −*y* direction to form directional radiation patterns.

**Figure 5:**
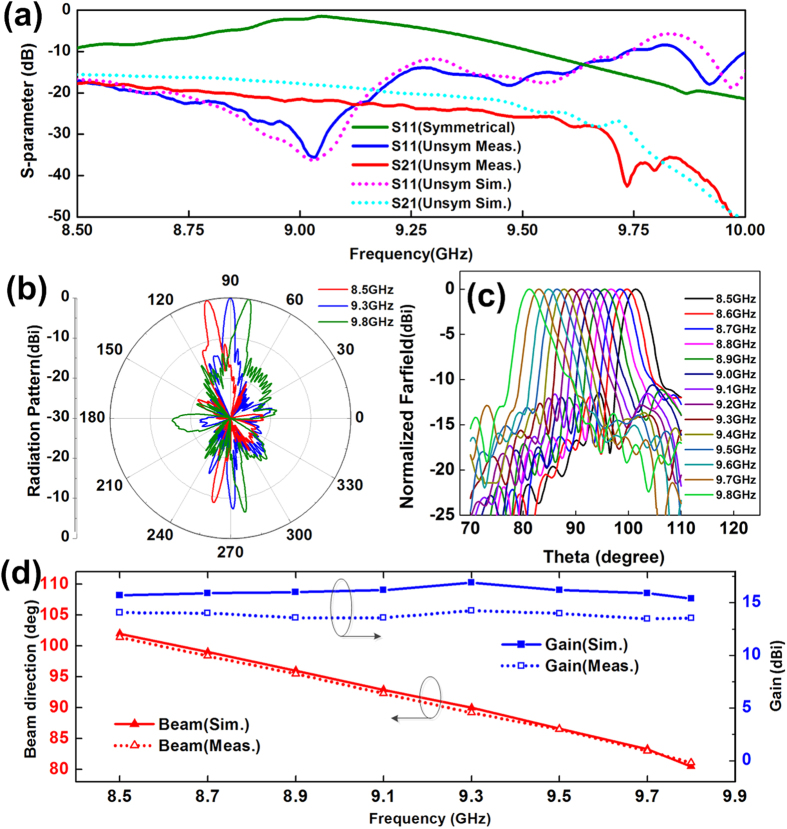
The measurement results of asymmetrically sinusoidally-modulated plasmonic waveguide. (**a**) The measured S parameters, which show that the reflection is reduced greatly by using asymmetrically plasmonic waveguide. (**b**) The measured far-field radiation patterns of E plane, which show that the main lobes are directed to 100°, 90° and 82° at 8.5 GHz, 9.3 GHz and 9.8 GHz, respectively. (**c**) The main lobe can steer from backward to forward, through broadside (90° at 9.3 GHz) continuously as frequency changes from 8.5 GHz to 9.8 GHz. (**d**) The measured gains and directions at different frequencies.

The measurement results show that both reflection coefficient (S_11_) and transmission efficient (S_21_) are lower than −10 dB from 8.5 GHz to 9.8 GHz, and the radiation efficiency is much larger than that of the symmetrical structure, as green line shown in Fig. 5(a). The measured far-field radiation patterns of E plane in the plane of *φ* = 90° (the *yoz* plane) are shown in Fig. 5(b), which clearly show that the main lobe of radiation directed to 100°, 90° and 82° at 8.5 GHz, 9.3 GHz and 9.8 GHz, respectively. The continuously scanning ability of leaky wave is demonstrated in Fig. 5(c), by changing the frequency from 8.5 GHz to 9.8 GHz, the main lobe of radiation can steer from the backward quadrant (100° at 8.5 GHz) to the forward quadrant (82° at 9.8 GHz), through the broadside direction (90° at 9.3 GHz) exactly, and the radiation efficiency is about 95% at broadside. The measured gains and radiation angles at different frequencies are demonstrated in Fig. 5(d), which show that the measured gain is about 13.5 dBi and the radiation angle can steer from 100° to 82° as the frequency changes from 8.5 GHz to 9.8 GHz. We notice that the gain is nearly unchanged in whole operating frequency region.

## Discussion

We presented a method to convert SSPPs to radiating waves using periodically-modulated plasmonic waveguide (i.e., corrugated metallic strip), whose surface impedance is modulated sinusoidally by changing the depths of corrugation grooves. The periodic modulation of the plasmonic waveguide introduces infinite space harmonics *k*_*N*_ (*N* = 0, ±1, ±2, …), in which the negative *N* corresponds to radiating fast waves under suitable conditions. In this paper, we choose *N* = −1 space harmonic to obtain single radiation beam. The periodically-modulated plasmonic waveguide is connected to CPW with 50 Ω impedance at two ports by matching transitions, which can make the plasmonic waveguide be easily integrated to conventional microwave circuits. We have fabricated and measured the sinusoidally-modulated plasmonic waveguide at microwave frequencies, and the measured results have good agreements with the numerical simulations. For symmetrically two-side plasmonic waveguide, we show that SSPPs are converted to radiating waves efficiently with omnidirectional radiation pattern in the azimuth direction while the radiation beam in the elevation direction is steered as the frequency changes. In order to improve the radiation efficiency at the broadside, we further designed an asymmetrical plasmonic waveguide with sinusoidal modulation, in which the metal grooves on both sides have *λ*/4 offset to each other. The measured results showed that the radiation efficiency is increased greatly by using the asymmetrical plasmonic waveguide, and the radiation pattern along azimuth is not omnidirectional any more. Both simulation and experiment results have demonstrated that the radiation beams can be steered from the backward quadrant to the frontward quadrant, passing through the broadside, which show good performance of the designed leaky-wave antenna.

## Additional Information

**How to cite this article**: Kong, G. S. *et al*. Continuous leaky-wave scanning using periodically modulated spoof plasmonic waveguide. *Sci. Rep.* **6**, 29600; doi: 10.1038/srep29600 (2016).
